# Heat stress and inadequate sanitary facilities at workplaces – an occupational health concern for women?

**DOI:** 10.3402/gha.v9.31945

**Published:** 2016-09-14

**Authors:** Vidhya Venugopal, Shanmugam Rekha, Krishnamoorthy Manikandan, Perumal Kamalakkannan Latha, Viswanathan Vennila, Nalini Ganesan, Perumal Kumaravel, Stephen Jeremiah Chinnadurai

**Affiliations:** 1 Department of Environmental Health Engineering, Sri Ramachandra University, Chennai, India; 2 Department of Biochemistry, Sri Ramachandra University, Chennai, India

**Keywords:** heat stress, sanitation facilities, genitourinary issues

## Abstract

**Background:**

Health concerns unique to women are growing with the large number of women venturing into different trades that expose them to hot working environments and inadequate sanitation facilities, common in many Indian workplaces.

**Objective:**

The study was carried out to investigate the health implications of exposures to hot work environments and inadequate sanitation facilities at their workplaces for women workers.

**Design:**

A cross-sectional study was conducted with 312 women workers in three occupational sectors in 2014–2015. Quantitative data on heat exposures and physiological heat strain indicators such as core body temperature (CBT), sweat rate (SwR), and urine specific gravity (USG) were collected. A structured questionnaire captured workers perceptions about health impacts of heat stress and inadequate sanitary facilities at the workplace.

**Results:**

Workplace heat exposures exceeded the threshold limit value for safe manual work for 71% women (Avg. wet bulb globe temperature=30°C±2.3°C) during the study period. Eighty-seven percent of the 200 women who had inadequate/no toilets at their workplaces reported experiencing genitourinary problems periodically. Above normal CBT, SwR, and USG in about 10% women workers indicated heat strain and moderate dehydration that corroborated well with their perceptions. Observed significant associations between high-heat exposures and SwR (*t*=−2.3879, *p*=0.0192), inadequate toilet facilities and self-reported adverse heat-related health symptoms (*χ*^2^=4.03, *p*=0.0444), and prevalence of genitourinary issues (*χ*^2^=42.92, *p*=0.0005×10^−7^) reemphasize that heat is a risk and lack of sanitation facilities is a major health concern for women workers.

**Conclusions:**

The preliminary evidence suggests that health of women workers is at risk due to occupational heat exposures and inadequate sanitation facilities at many Indian workplaces. Intervention through strong labor policies with gender sensitivity is the need of the hour to empower women, avert further health risks, and also enhance productivity for the few million women workers who contribute largely to the country's economy.

## Introduction

India is a tropical country with a very hot summer season in many parts that threatens the health of millions of people (1, 2), and workers are exposed to excessive workplace heat (3, 4). In 2013, more than 600 deaths were reported due to heat waves in the southern Indian states Andhra Pradesh and Odisha, where the temperatures have soared as high as 47.2°C, while many other states experienced severe drought (2), and excess mortality due to heat was reported in Ahmedabad (1). During the hottest season in parts of India, daily climate conditions are untenable with respect to physical labor (5, 6). Hot work environments increase the health risks for poor workers engaged in manual work and reduce their hourly productivity and daily incomes (4). One of the most direct health effects occurring from global change in temperatures is the increase in mortality and morbidity associated with exposure to high ambient temperatures in certain parts of the globe, including parts of India (7).

Heavy workload in hot working environments creates substantial surplus heat inside the body, and when the air temperature exceeds 37°C, evaporation of sweat becomes the only mechanism to cool the body, but sweat evaporation is strongly impaired by high air humidity and clothing (8). Many jobs involving high-heat exposure, especially in informal sectors, have a large proportion of women workers who are engaged in physical work (9). Women, because of their size, physical capacity, and other physiological factors, are more vulnerable to health risks (10–12). Women in developing nations have to handle the burden of housework and heavy manual work in jobs which is sometimes beyond their physical capacity (13), and when combined with hot working environments further increases the health risks (14, 15). Also, Indian women could be more vulnerable to elevated environmental temperatures as evidenced by a higher number of reported excess deaths of women during the heat wave in Ahmedabad, 2010 (1).

Individuals exposed to high temperature at their workplace suffer from several heat-related illnesses, including heat stroke, heat exhaustion, heat cramps, heat syncope (fainting), and heat rash (16–22). In such conditions, sweat output often exceeds water intake, resulting in a body water deficit (hypohydration) and electrolyte losses (23). Dehydration and lack of adequate fluid replacement can undermine the health and performance of an individual and leave them particularly vulnerable to the ill effects of high-heat loads (24, 25) that can further be aggravated by lack of proper sanitation facilities at workplaces.

No issue touches the lives of women, particularly working women, as intimately as that of access to proper sanitation. Although men also suffer from the burden of poor sanitation, they are more likely to resort to other means to relieve themselves. But women's anatomy, modesty, and susceptibility to attack do not allow them to discreetly relieve themselves in public places (26–28). Lack of adequate sanitation facilities at the workplaces subjects women to suffer periodically from urogenital problems such as urinary tract infection (UTI), burning sensation, and edema/swollen legs (29).

Lack of access to toilets in some workplaces, in turn, causes some women to eat and drink less, in order to avoid having to defecate or urinate for several hours, thus putting them at a higher risk for malnutrition (3). The most commonly provided heat protection advice to avert adverse health impacts of heat exposures, among others, is to wear lightweight loose-fitting clothing, seek out an air-conditioned or cool environment, avoid physical activities, and drink fluids regularly (7). Drinking frequently to maintain the body's water balance is possible but might create another issue of need to urinate frequently, which becomes an inconvenience to women workers in jobs with lack of access to proper toilet facilities at their workplaces (30). The health burden of inadequate sanitation may cause women to purposely restrict their fluid intake, despite thirst or heat signals, in order to avoid the need for a toilet (31, 32). Delaying urination and refraining from drinking water to avoid using the toilet often lead to dehydration in heat and cause internal injuries such as acute kidney injuries (AKI) or UTIs (28). For women who are menstruating, the need for adequate sanitation becomes even more acute (30), and the lack of access to it puts them at a higher risk of urogenital infections (4).

Intergovernmental Panel on Climate Change has predicted increase in temperatures by the year 2100 to the extent of 2–3°C around the globe according to RCP scenario 6.0 (33) and is expected to affect the living and working environments that could create health threats for millions of people (34). Although the rates of heat-related morbidities and mortalities are declining, and future trends look better in many countries (35, 36) and in many developing countries with high ambient temperatures, the burden of health risks due to heat is still looming (7, 14, 37, 38). The heat situation might be further aggravated in many workplaces with minimal/no cooling intervention that is very common in developing nations and for outdoor women workers performing manual work, especially with the predicted rise in temperatures due to climate change (33).

Importantly, many jobs involving high-heat exposures and heavy manual workload in informal sectors, like in construction, brick kilns, and agriculture, have a large proportion of women workers in India, (9) and these workplaces lack basic facilities like toilets, or most often have none. As per the 2012 census, the total population of India (2012) was 1.22 billion out of which 591.4 million are females. The number certainly indicates that these people are invaluable assets to the country, thus making it necessary to empower them to contribute to the nation's economy. Hence, poor health of working women is a concern at both an individual and national level.

In such context, it becomes important that studies addressing the issue in question are conducted to bring in the research understanding, knowledge, and evidence to help build upon existing labor policies that can be implemented as effective prevention policies to help avert current and future health risks for millions of poor women workers. No study has been conducted in Indian workplaces to understand the combined influence of heat exposures and inadequate sanitation facilities and associated health risks to women workers. The investigators thought it was imperative to address the issue and conducted the study in three occupational sectors in southern India.

## Methodology

We conducted this cross-sectional study in three occupational sectors: brick manufacturing (two brick kilns in two districts of Tamil Nadu), one steel manufacturing industry, and agriculture fields (five agricultural fields in two different states in southern India) in 2014–2015. These sectors employed predominantly women for certain types of jobs which involved manual hard work. The women workers had direct heat exposures throughout the day and had long working hours, depending on the season, in brick and agriculture. We obtained prior ethical clearance from the Institutional Ethics Committee and permission from the concerned management for the study. Our qualified and experienced nurse explained the risks and benefits of participating in the study to the workers.

### Data collection using questionnaire

We recruited and interviewed 312 women workers engaged in medium-to-heavy work who volunteered for the study to collect the necessary information after getting informed consent from the workers. We administered a modified version of an internationally validated High Occupational Temperature Health and Productivity Suppression questionnaire (Supplementary file) (4) to collect the information on age and sex, sociodemographics, personal habits, workload, clothing worn, worker's time-activity pattern, toilet availability and use, menstrual cycle phase, health history, information on intake history of any medication, and occupational history at the workplace. An elaborate section on self-reported heat-related health illnesses; information pertaining to existing sanitation facilities, in particular toilet facilities at workplace available to women employees; behavioral patterns; water intake, heat stress, and workload; and their perceptions on the health impacts of heat stress and lack of toilet facilities that reflected their personal experiences of the heat and the symptoms of each illness were explained to the study participant by the interviewer. Most of the workers could speak and understand the local language that was spoken by the interviewer.

### Heat exposure assessment and physiological measurements

Occupational heat exposure at each participant's workplace was assessed as per the protocols recommended by NIOSH, USA (1986) (39). Quantitative data on environmental heat stress within the industry were assessed via measurements of the wet bulb globe temperature (WBGT) using a calibrated portable heat stress monitor (QuesTemp°34; QUEST Technologies, USA) during the hottest part of the day (10 a.m.–4 p.m.). We allowed the equipment to stabilize for 15 min before taking measurements (40) in each location and kept away from objects that might block radiant heat or air flow. The work category of the workers was based on the judgment by a trained industrial hygienist based on ACGIH guidelines and by observations that were compared with the ACGIH screening limits (40).

Data on physiological parameters, such as core body temperature (CBT), sweat rate (SwR), and urine specific gravity (USG), were assessed for women employees who volunteered and consented for such investigations (*n*=177) during the work shift. Care was taken not to hamper their workflow, as the participants did not receive any compensation for participating in the study. CBT was assessed using 3M, Quest Temp Personal II monitors, which give minute-by-minute CBT variations experienced by the worker. It was measured before the onset of work (pre-exposure CBT) and after the work shift (post-exposure CBT). The reference range for the workers was increased greater than 1°C in post-exposure CBT (41).

The SwR was calculated using the formula of Canadian Sports Association (42). The body weight was measured, using a weighing scale (accurate to 0.1 kg) before the start and end of the work. A recommended limit value for SwR is 1.0 L/h (41). Data on the quantity of liquid consumption during the study time in a work shift and the time gap between urinations were collected from the study participants to check the SwR. USG was measured via a standard urinometer, and the safe limit of USG was considered as 1.010–1.020 (43).

Data analysis was done using basic statistical tools such as Excel and R software. For the descriptive data, single proportions like prevalence of heat stress and heat-related health problems/productivity loss were tested for significance using the *t*-test, chi-square test, and odds ratio (OR). A standard cutoff of 0.05 was used to interpret the significance of the *p*-values for all analysis.

## Results

The mean age of the study population (312 female workers) from three occupational sectors, brick manufacturing, steel manufacturing, and agriculture fields, was 42±11.5 years, with only 30% having some basic level of education and all other were illiterates. Twelve percent had some pre-existing medical conditions, such as diabetes and hypertension. Most of the participants from agriculture fields and brick manufacturing were engaged in a similar profession or similar type of work for more than 10 years. The participants from the steel manufacturing industry were engaged in the same profession of housekeeping and cleaning, which was physically intense for an average of 8 years and had chronic exposures to high heat at their workplace.

### Heat stress profile of the workplace

**Fig. 1:**
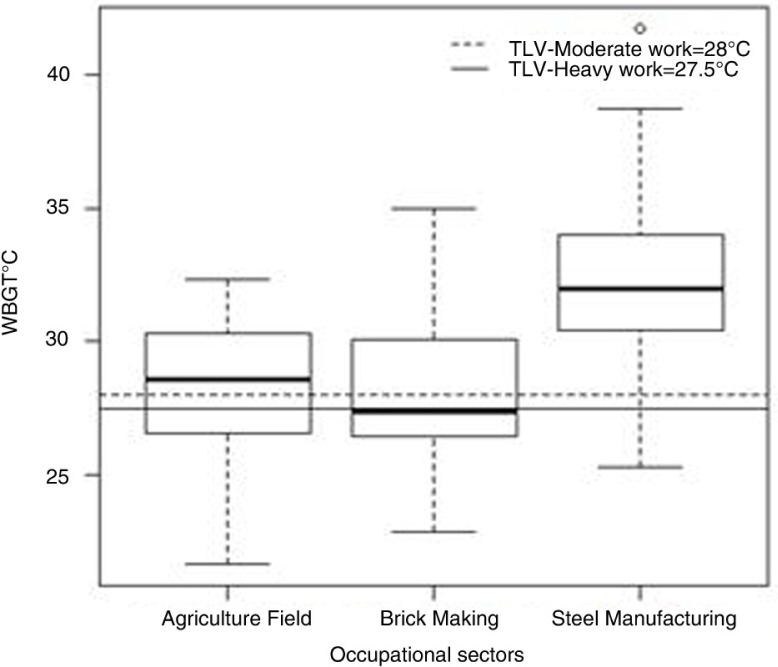
Distribution of WBGT measurements in 312 female workers in brick manufacturing, steel industry, and agricultural fields (2014–2015).

**Fig. 2:**
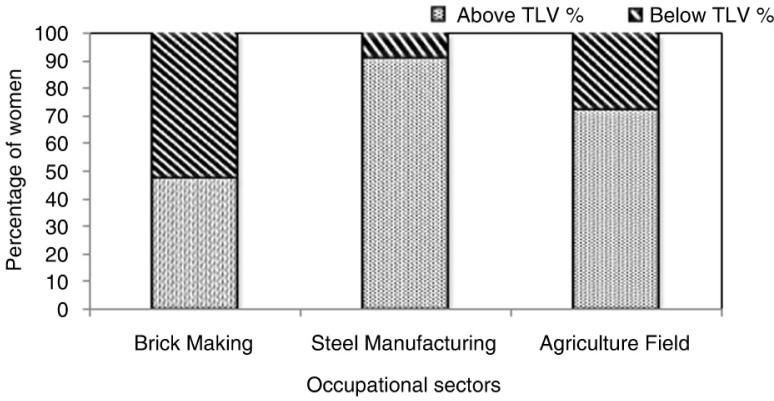
Distribution of workers (%) working above and below threshold limit values (TLVs) in brick manufacturing, steel industry and agricultural fields (2014–2015) *n*=312.

The results of the area WBGT measurements reflect the heat exposures of the 312 workers (Fig. 1), and it is apparent that about 71% (*n*=222) of the workers had WBGT exposures higher than the recommended threshold limit value (TLV) as per ACGIH guidelines (40). Forty-two percent of the workers’ self-reported perceptions were that high-heat exposures throughout the year was not an uncommon phenomena in their workplace, with significantly higher heat exposures during summer season. Heat exposures in various work categories show the percentage of workers working above and below the safe TLV limits in the study population (Fig. 2).

### Heat stress and health impacts

Of the 312 workers recruited for the study, 95% of the women workers perceived that heat stress exposures had adverse impacts on their health and well-being (Table 1), and widespread concerns about heat-related health issues were prevalent among the women workers. Nearly 73% of these workers reported that because of heat exposures they have experienced chronic fatigue, 44% suffered from headache, and ~87% reported excessive sweating and thirst during work shifts. Their perceptions are supported by the measured physiological indicators of heat strain that are presented in Table 2. A significant positive association was observed for the increase in SwR of the workers (7%), the first indication of dehydration, for those women (*n*=123) whose heat exposures were above the TLV limits (*t*=−2.38, *p*=0.0192).

**Table 1:** Distribution of heat stress exposure profiles and worker perceptions of heat-related health impacts resulting from occupational heat stress in select occupational sectors in southern India (2014–2015)

S. no	Occupational sectors	Heat exposures WBGT (°C) Mean±SD	Perceived heat-related health impacts % (frequency)
1.	Brick industry (*n*=67)	28.2±2.7	99 (*n*=66)
2.	Steel industry (*n*=87)	32.3±3.4	89 (*n*=77)
3.	Agriculture (*n*=158)	28.0±2.9	97 (*n*=153)

Note: *n*=sample size.

**Table 2:** Distribution of measured physiological heat strain indicators among workers from select occupational sectors in southern India (2014–2015)

		Percentage of women with higher than recommended limits in physiological heat strain indicators (%)					
		
S. No.	Work sector	CBT(°C) >1°C	Mean±SD	SwR (L/h) >1.0 L/h	Mean±SD	USG >1.020	Mean±SD
1.	Brick industry (*n*=29)	17.2	0.6±0.5	13.8	0.5±0.4	6.9	1.014±0.005
2.	Steel industry (*n*=85)	1.2	0.4±0.3	4.7	0.4±0.2	17.6	1.012±0.013
3.	Agriculture (*n*=63)	11.1	0.5±0.4	1.6	0.4±0.2	6.3	1.010±0.005

### Sanitation facility and health impacts

Women employed in the steel industry and a few agriculture workers in one out of the five agricultural fields surveyed (in the outskirts of Chennai city) had access to public toilets facilities nearby (<0.5 km). However, the women workers in both the brick kilns and other agricultural fields (that were remotely located) did not have toilet facilities and had to resort to open fields for reliving themselves. Sixty-four percent (*n*=200) of the study participants did not have access to toilet facilities at their workplaces, and the association between women reporting heat-related health symptoms and lack of access to toilets at workplaces was significant (*χ*^2^=4.0397, *p*=0.0444). Among the women who had no access to toilets, 174 women (87%) reported having had to experience specific genitourinary problems periodically, which was also significantly associated (*χ*^2^=42.92, *p*=0.0005*10^−7^) (Table 3). The women without access to toilets had six times higher odds of prevalence of genitourinary issues compared with the women with access to toilets, such as in the steel industry (OR=6.01, 95% CI: 3.45–10.47 *p*<0.0001) (Table 3). Women who had no access to toilets at workplaces also reported drinking less water (5%, *n*=17) to avoid having the need to frequently use the toilet (*χ*^2^=4.76, *p*=0.0290), and a significant association was observed between ‘not drinking water’ (<1 L/shift) and prevalence of reported ‘genitourinary issues’ (*n*=17) (*χ*^2^=20.03, *p*=0.0076*10^−04^). It was also observed that women ‘not drinking sufficient water’ had four times higher risk of developing genitourinary issues compared with women ‘drinking sufficient water’ (OR 4.01, 95% CI: 2.16–7.45, *p*<0.0001) (Table 3).

**Table 3:** Relationship between lack of access to toilet facilities on self-reported health outcomes, fluid intake pattern, and ‘withholding’ behavioral modification of women workers from brick manufacturing and agricultural fields in southern India (2014–2015)

S. No.	Lack of toilet facilities versus study variables	Sample size	No. of workers reporting with symptoms Frequency (%)	*χ*^2^	*p*	*df*
1.	Inadequate fluid consumption	312	17 (5.4)	4.762	0.0291	1.0
2.	‘Withholding urine’		24 (7.7)	12.22	0.0003	1.0
3.	Genitourinary issues		174 (55.8)	42.928	0.0006×10^−7^	1.0

	Study variables	Sample size	Workers reporting genitourinary issues Frequency, (%)	Odds ratio	*p*	95% CI

1.	Lack of toilets versus genitourinary issues	312	174 (55.8)	6.0	0.0001	3.45–10.47
2.	Inadequate fluid consumption versus genitourinary issues		17 (5.4)	4.0	0.0001	2.17–7.46

**Fig. 3:**
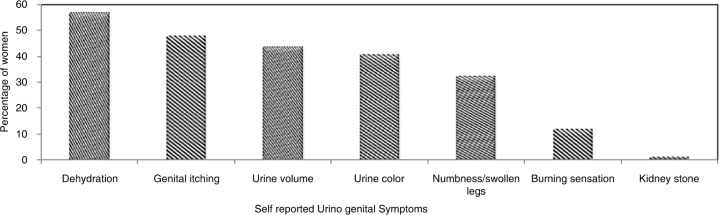
Self-reported urogenital symptoms due to lack of access to toilet facilities by women workers working (*n*=200) in brick manufacturing and agricultural fields (2014–2015).

Women without access to toilets (*n*=200) also reported changes in urine volume (44%), color (39%), experiencing frequent recurring burning sensations, and UTIs (11%). Behavioral modification of ‘withhold from urinating’ than it is convenient (about 2–4 h) was adopted by the women (*n*=24), which was significantly associated with lack of access to toilets (*χ*^2^=12.2, *p*=0.0003). A small percentage (2.0%) of women workers also reported being diagnosed and treated for kidney stones (Fig. 3). Ten percent reported staying home from work during their menstrual period for hygiene purposes, with consequent loss of wages for the day and psychological stress of displeasure of the supervisors.

## Discussion

This study demonstrates that women workers are and can be exposed to high levels of heat at work, which can potentially make them vulnerable to heat-related illnesses, especially during hot seasons.

### Heat exposures and associated health risks

Studies point to increased rate of mortality and potential morbidity among the workers exposed to high ambient temperature (43–50). In this study, about 71% of the women were exposed to high occupational heat stress and were working above the safe threshold limit of exposure (40). The maximum WBGT of 41.7°C was found in the steel industry (Table 1) where the women were exposed to high radiant heat from processes like smelting, casting, and furnaces. Because the women in the steel industry were engaged in housekeeping and cleaning in the manufacturing area and outdoors, they had exposures to radiation from both the sun and the various manufacturing processes throughout their shifts. Such extreme heat exposures can make the women workers more vulnerable to the risks of developing heat-related illnesses (51, 52). Heat-related health issues were reported the highest in brick and agriculture sectors with more than 95% of the workers experiencing adverse health issues due to heat stress. The women in the open fields, like in brick and agriculture, felt ‘very exposed’ to many adversities and had to fend for themselves in case of medical emergencies related to heat. The distance of the work locations from the nearest hospital or doctor's clinic, which was usually quite far from the farms/brick kilns, was a big concern for the workers. In the steel industry, although 89% of women workers reported adverse impacts of heat on their health (Table 1), only 40% reported productivity loss, as there were very few options for the women workers to self-pace their work due to tight production targets. Clearly, the women were pushing beyond their physiological limits to complete the set target for the day. Working at high temperatures and beyond work capacity, without appropriate work rest ratios, subjects the workers to the risks of developing heat-related illnesses (26, 41). Although the women in the steel industry were working beyond their work capacity and were exposed to much higher WBGTs than in the brick and agricultural fields, they are partially protected by insurance and have an occupational health center in case of occurrence of any heat-related health issues that creates a ‘psychologically safe’ feeling to the workers.

### Physiological response to heat stress

The manifestations of heat exposures are usually expressed by the physiological responses, viz., rise in CBT, SwR, and USG, and the relationship between physiological indicators of heat stress and the associated adverse health risks are well established (41). The behavioral modifications by the women in fluid intake and urinating pattern due to lack of access to toilets also influence the physiological indicators resulting in consequent health concerns (38). The significant association observed among the women who were exposed to heat stress (above TLVs) and increased SwR > 1 L/h (*n*=9) (*t*=−2.38, *p*=0.0192) clearly indicates the progression toward cellular dehydration (41). The percentage of women in the brick sector having SwR > 1 L/h (14%) and CBT rise above >1°C (17%), compared with steel industry and agriculture (Table 2), could be attributed to the exposures to intense heat from the open furnaces in the brick kilns and heavy workload in addition to the inadequate fluid intake due to lack of toilets at work. Although the women in the steel sector were exposed to higher WBGTs compared with women in agriculture and brick kilns, the metabolic workload was partially compensated by automation that reduced their work intensity. In spite of the sanitation facilities available in the steel industry, the women with USGs > 1.020 were higher (18%) in the steel sector compared with the brick and agriculture sectors, which could be a result of chronic high-heat exposures (radiant heat) from the furnaces. Research has shown that excessive sweating and consequent dehydration raises heat strain (53) and increases the risk of developing heat illness (47, 54). The increase in SwR can be affected by a number of factors that include temperature, heat acclimatization, and type of clothing worn, (55) especially for some of the women participants who wore multiple layers of clothing driven by culture and tradition which had an adverse influence on the heat load imposed on them while working as it impedes evaporative cooling (56).

### Inadequate sanitation facilities and associated risks

In a scenario with lack of access to toilets, the behavioral modifications in fluid intake and urination pattern of the women may further increase the risks (32). In this study, the results of the USG measurement indicate that about 12% of the study population with USG > 1.020 may be dehydrated, a theory that is also strongly supported by self-reported symptoms suggestive of dehydration (56%). Reasons that could be attributed for this finding are that the women did not drink enough fluids to compensate for the sweat loss due to continuous high-heat exposures, heavy physical work, and behavioral modification in fluid intake to avoid using toilets. USG rises with the decrease in body mass caused by sweat loss and dehydration induced by heavy physical activity (57–59), and such chronic and prolonged dehydration will potentially subject the workers to genitourinary issues including decreased kidney functioning/kidney anomalies (60). Dehydration and volume depletion have been cited among major causes of UTIs, AKI, (61) and increased cardiovascular strain during heat stress (62).

The women workers, who had no access to proper toilet facilities at their workplaces (agriculture and brick sectors), had reported more heat-related symptoms (*n*=194) (*χ*^2^=4.03, *p*=0.0444), including dehydration. Water intake was minimized, and signals of thirst were ignored by the women consciously to avoid frequenting the need for using a toilet. The significant association seen between lack of access to toilets and self-reported genitourinary symptoms (*n*=174) (*χ*^2^=42.92, *p*=0.0005*10^−7^) stands as evidence that lack of sanitation facilities is a big concern for the women and important for their good health (63).

### Importance of sanitation facilities at the workplace

Irrespective of the profession, women – whose anatomy and modesty do not allow them to relieve themselves in public – have no choice but to wait to relieve themselves discreetly or until dark, when there is less risk of being seen or accosted (64) (which is common practice in rural settings). For protecting women's safety, dignity, and self-respect, privacy for sanitation is essential (15, 65) in any setting, home, or work. Studies show that women eat less and even drink less to make it easier to ‘hold it’ for a long time (63), but are unaware that by doing so they may be subjected to more health risks in future, which is clearly evidenced in this study (Table 3). The lost wages due to being away from work that was reported by ~10% of the women during their menstrual period to avoid experiencing anxiety of personal hygiene due to lack of access to toilets will potentially affect their economic status (21) and increase the psychological stress of discontentment of their employers. Interventions such as improving the basic sanitation facilities and reducing heat exposures by simple techniques will improve health equity because low-income people generally end up working in jobs in the unprotected informal sector with the greatest health risks (66). It is essential to urgently address issues, such as lack of access to toilet facilities at workplaces, which add to the risk already posed by hot climates for working people. Good access to toilet facilities for women at the workplace reduces anxiety and improves the ability to do the work properly, safely, and with concentration, even during their menstrual cycle (67).

Although heat and lack of toilets can affect every worker, the special occupational health and safety needs of women workers have to be met because they are ‘vulnerable workers’ as classified by the International Labor Organization (68). Moreover, the expectation for women to have basic sanitation facilities such as a ‘toilet’ at the workplace to maintain one's dignity is not unreasonable. Meeting this expectation is a ‘win–win’ situation for the employee and the employer, with public health benefits. Providing comfortable basic facilities at workplaces will help attract women for employment, reduce absenteeism, improve the health and welfare of the working women, and protect the public from nuisance and hazards of open defecation. Dialogs and cooperation between the public health authorities, non-governmental organizations, women's groups, employers, and the labor ministry will set the stage for the needed change for improved occupational health of the women in the country.

### Strengths and limitations of the study

1. This is the first study to explore the influence of occupational heat exposures and access to toilet facilities on women's health at workplaces in Indian occupational settings.
2. The findings of the study have important preventive policy implications for developing countries with hot climates such as India, where millions of working women are exposed to the risks of heat stress and lack of access to toilets at work.
3. The main weakness of this study is that there is no control group (both with respect to heat exposures and toilets) to determine if the prevalence is different in the non-exposed group.
4. No other supportive quantitative data or clinical records as evidence of health impacts could be collected from the workers due to unavailability of those records.
5. This sample is only a very small proportion of the entire population of working women in the country. The findings from the study, therefore, cannot be generalized.

## Conclusions

The study demonstrates that women workers who are exposed to high-heat environments and inadequate sanitation facilities at Indian workplaces have significant risks of heat-related health illnesses and urogenital issues. This study draws attention to the public health concern that many workplaces in developing nations still lack basic sanitation facilities such as toilets. This, combined with the predicted trends in temperature rise associated with climate change, could further exacerbate the health risks for the women, which merit attention. Intervention studies and further research to strengthen the evidence are urgently needed to translate the research into effective prevention policies by joint efforts of the public health authorities, industries, and labor ministry. Designing comprehensive gender-sensitive labor policies and workplace interventions are the need of the hour in order to avert health risks for millions of women workers, especially in the developing nations with hot climates.

## Supplementary Material

Heat stress and inadequate sanitary facilities at workplaces – an occupational health concern for women?

## Paper context

Heat stress and inadequate sanitation facilities are common risk factors in workplaces in developing countries with hot climates. Although the impact of heat on health is well established, the effects of these risk factors on women workers remain unexplored. Results of this investigation stand as preliminary evidence of the significant adverse health implications of these risk factors on the women workers. Urgent gender-sensitive policy interventions could avert future health risks and empower women.
