# Supplementary material for: Heat stress and inadequate sanitary facilities at workplaces – an occupational health concern for women?
Source: Glob Health Action. 2016 Sep 14;9:10.3402/gha.v9.31945. doi: 10.3402/gha.v9.31945 (PMC5025522; doi:10.3402/gha.v9.31945)
Supplement: Heat stress and inadequate sanitary facilities at workplaces – an occupational health concern for women? [file GHA-9-31945-s001.docx]

**Supplementary file:**

**QUESTIONNAIRE**

**Part 1 General information about person interviewed and the organization she/he represents**

1. Name:
2. ID No:
3. Date of interview:
4. Name of the interviewer:
5. Name of the industry:
6. Location of the industry:
7. Type of industry:
8. Age:
9. Age group: 🗖_1_ 10-20 /🗖_2_ 21-30/ 🗖_3_ 31-40/ 🗖_4_ 41-50/ 🗖_5_ 51-60/ 🗖_6_ 61-70
10. Sex: 🗖_1_ Male / 🗖_2_ Female
11. Education: 🗖_1_ Illiterate / 🗖_2_ Primary / 🗖_3_ Secondary / 🗖_4_ Higher Secondary /

🗖_5_ University/ 🗖_6_ Polytechnic/Diploma

1. Designation: 🗖_1_ Worker 🗖_2_ Supervisory 🗖_3_Manager
2. Worker category:
3. Smoking: 🗖_1_ Smoker /🗖_2_ Non Smoker /🗖_3_ Ex smoker
4. Consuming alcohol: 🗖_1_ Yes 🗖_2_ No 🗖_3_ Ex
5. Any existing illness: 🗖_1_Diabetes 🗖_2_Hypertension 🗖_3_Respiratory illness, specify 🗖_4_Others, specify 🗖_5_None

**Part 2 Questions concerning the type of work**

1. Type of work: 🗖_1_Light 🗖_2_Moderate 🗖_3_Heavy 🗖_4_ Very Heavy
2. Do you work near a direct heat source(naked flame/hot air/outdoors/radiant heat) 🗖_1_ Yes / 🗖_2_ No
3. Is the place you work well-ventilated? 🗖_1_ Yes / 🗖_2_ No
4. How many ***hours*** per day do you usually work excluding regular break timings? _________
5. Do you have additional breaks during summer? 🗖_1_ Yes / 🗖_2_ No
6. If yes, mention no. of ***hours/minutes***_________________
7. How long you are employed here? ____________***years/months*** (more than 6months means acclimatized)
8. What was your previous job and where (relating to temp)? ____________

***Part 3 Questions in relation to heat exposure at work***

1. Are you comfortable with the ambient temperature? 🗖_1_ Yes / 🗖_2_ No
2. Is heat exposure a problem during the hot season? 🗖_1_ Yes / 🗖_2_ No
3. How many months do you feel hot /uncomfortably hot in this workplace?

🗖_1_ 1-3 months/ 🗖_2_ 4-6 months/ 🗖_3_ 7-9 months / 🗖_4_ 9-12 months/ 🗖_5_ Never

1. Describe how bad the heat stress can be in the hot season.

🗖_1_ Extremely bad/ 🗖_2_ Very bad/ 🗖_3_ Bad/ 🗖_4_ Manageable/ 🗖_5_ No stress at all

***Part 4 Questions concerning impacts of heat on health***

1. Have you ever had these symptoms at work?
2. Excessive sweating 🗖_1_ Yes / 🗖_2_ No
3. Muscle/Heat cramps 🗖_1_ Yes / 🗖_2_ No
4. Thirst 🗖_1_ Yes / 🗖_2_ No
5. Tiredness/weakness/🗖_1_ Yes / 🗖_2_ No
6. [dizziness](http://www.medicinenet.com/script/main/art.asp?articlekey=97800) 🗖_1_ Yes / 🗖_2_ No
7. headache 🗖_1_ Yes / 🗖_2_ No
8. [nausea or vomiting](http://www.medicinenet.com/script/main/art.asp?articlekey=41943) 🗖_1_ Yes / 🗖_2_ No
9. [fainting](http://www.medicinenet.com/script/main/art.asp?articlekey=1945) 🗖_1_ Yes / 🗖_2_ No
10. Prickly heat 🗖_1_ Yes / 🗖_2_ No
11. Heat stroke 🗖_1_ Yes / 🗖_2_ No
12. Others__________________________

***Part 5 Questions concerning impacts of heat on worker’s productivity***

1. Have you ever taken sick leave/permission due to heat? 🗖_1_ Yes / 🗖_2_ No
   1. If yes, approx. how many ***hours/days*** in a ***week/month***?_________________
2. Have you lost any wages due to absenteeism in summer months? 🗖_1_ Yes / 🗖_2_ No
   1. If yes, how much _Rs._________________________?(currency)
3. Have you ever been advised/ ever been admitted in hospital/medical centre to take off due to heat related illness? 🗖_1_ Yes / 🗖_2_ No
   1. If yes, approximately how many ***days***__________________________
4. How does heat affect other aspects of your work (during hot seasons)

🗖_1_ Absenteeism/ 🗖_2_ Less productivity/ 🗖_3_ Irritation/Interpersonal issues/Work related issues with manager/ 🗖_4_ Take more time to complete same task

1. Do you have production target? 🗖_1_ Yes / 🗖_2_ No
   1. If Yes, how much?__________________________
2. Do you complete your production target? 🗖_1_ Yes / 🗖_2_ No
   1. If no, how much target is not completed? __________________________units
3. To achieve production target or complete work do you have to work extra hours?

🗖_1_ Yes / 🗖_2_ No

- 1. If yes, how many extra hours __________________________

***Part 6 Questions concerning impacts of clothing on heat stress and productivity***

1. Dress material of the workers (Indian equivalent to ACGIH)

🗖_1_ Breathable cotton/ 🗖_2_ Thick cotton overall/ 🗖_3_ Rayon/Nylon/

🗖_4_ Plastic PPE/ 🗖_5_ Others______________________

1. Worker perception about dress Material of the worker:

🗖_1_Comfortable/ 🗖_2_ Moderately comfortable/ 🗖_3_ Uncomfortable/ 🗖_4_ Others____

1. If uncomfortable, can you give any suggestions to improve? __________________________
2. Do you feel hotter with uniform/ Does the Dress Material increase heat stress/comfort? 🗖_1_ Sure/ 🗖_2_ Maybe/ 🗖_3_ Not sure/ 🗖_4_ No, not at all
3. Does clothing reduce your work output?

🗖_1_ Sure/ 🗖_2_ Maybe/ 🗖_3_ Not sure/ 🗖_4_ No, not at all

***Part 7 Questions concerning coping mechanisms***

1. How do you limit heat exposure/cope with heat exhaustion, when needed?

🗖_1_ take rest/ 🗖_2_ Change/remove clothing/ 🗖_3_ drink water/ 🗖_4_ cool shower, bath, or sponge bath/🗖_5_ move to an air-conditioned/cooler environment/ 🗖_6_ any other method, do specify____________________

1. Is sufficient water available at all times when you need it? 🗖_1_ Yes / 🗖_2_ No
2. Do you drink water at work? 🗖_1_ Yes / 🗖_2_ No
   1. If yes, how much? ________________(litres)
3. Do you take any traditional special diet to cope with heat? 🗖_1_ Yes / 🗖_2_ No
4. What traditional or other methods do you adopt for coping with heat? ________________________________________
5. Do you spend more money during hot seasons to cope with heat? 🗖_1_ Yes / 🗖_2_ No

a. If yes, specify ____________________ (Currency units)per week/month

1. Do you spend more time to cope with heat? 🗖_1_ Yes / 🗖_2_ No
   1. If yes how much_________________(min/hrs) – convert to % of productivity time or personal time
2. Does the time spent on coping heat impact your social life? 🗖_1_ Yes / 🗖_2_ No
3. How does it affect your social life? 🗖_1_ Moderately/ 🗖_2_ Highly/ 🗖_3_ Extremely/ 🗖_4_ No impact

***Part 8 Questions concerning access to drinking water***

1. Do you have access to drinking water at your work? 🗖_1_ Yes / 🗖_2_ No
   1. If yes: From where do you get that water?
2. From a water tank
3. Water bottles
4. A river or lake etc. close to the area
5. Others
6. Do you drink when you feel thirst? 🗖_1_ Yes / 🗖_2_ No
   1. If no: why not?
7. Not access to water
8. Frequent urination and no access to toilets
9. It disturbs work continuity
10. Others
11. Do you think you would drink more if you had better access to toilets? 🗖_1_ Yes / 🗖_2_ No
    1. If no: Why not?
12. The access to water is not good
13. The work does not allow it
14. Others

***Part 9 Questions concerning kidney problems***

1. Have you noticed changes in your urine volume? 🗖_1_ Yes / 🗖_2_ No
2. Do you have excessive tiredness or skin itching? 🗖_1_ Yes / 🗖_2_ No
3. Do you have numbness or swollen legs or hands due to water retention?

🗖_1_ Yes / 🗖_2_ No

- 1. If Yes to 1, 2 and 3, what is the color of your urine?
     1. Reddish
     2. Dark Yellow
     3. Yellow
     4. Colorless
     5. Have not noticed
     6. Don’t know

1. Have you been treated for kidney stones? 🗖_1_ Yes / 🗖_2_ No
   1. If yes: When? -----------------------------------------------
2. Do you feel pain in the bottom of your back? 🗖_1_ Yes / 🗖_2_ No
   1. If yes: For how long?
      1. The last week
      2. The last month
      3. The last 2-6 month
      4. Longer than the last 6 months
3. Do you think this problem would have been better if you had better access to toilets? 🗖_1_ Yes / 🗖_2_ No
4. Have you ever been admitted to hospital/medical centre because of kidney problems? 🗖_1_ Yes / 🗖_2_ No
   1. If yes: Approximately for how many days?.................................................................

***Part 10 Questions concerning dehydration***

1. Do you feel dehydrated? 🗖_1_ Yes / 🗖_2_ No
2. Do you have any pressure ulcers? 🗖_1_ Yes / 🗖_2_ No
3. Do you have any other skin conditions? 🗖_1_ Yes / 🗖_2_ No
4. Do you have nausea or fainting spells? 🗖_1_ Yes / 🗖_2_ No
5. Have you ever been admitted to hospital/medical centre due to dehydration?

🗖_1_ Yes / 🗖_2_ No

- 1. If yes: Approximately for how many days?.................................................................

1. Do you think this problem would have been better if you had better access to drinking water facilities/toilets? 🗖_1_ Yes / 🗖_2_ No

***Part 11 Questions concerning access to toilets***

1. Do you have access to toilets at work? 🗖_1_ Yes / 🗖_2_ No

***Part 12 Questions if you have access to toilets***

1. Do you use the toilets? 🗖_1_ Yes / 🗖_2_ No
   1. If yes: How often
2. 1-2 times per day
3. 3-4 times per day
4. 5-6 times per day
5. >6 times per day
   1. If no: Why not
6. They are too far away
7. Difficulties to get away from work
8. They are not clean/in good hygienic condition
9. Others
10. Can you talk about it in the open? 🗖_1_ Yes / 🗖_2_ No
    1. If no: Why not
11. It makes you feel uncomfortable
12. It is taboo
13. People around you would treat you outrageous
14. Others

***Part 13 Questions if you don’t have access to toilets***

1. How often do you go to urinate and defecate?
2. 0 times
3. 1-2 times
4. 3-4 times
5. 5-6 times
6. >6 times

If b-e:

1. Where do you go to urinate and defecate?
2. An hidden area (small building etc.)
3. In the forest
4. On the streets
5. Others
6. Do you go alone? 1Yes / 2No
7. Do you feel comfortable with that option? 🗖_1_ Yes / 🗖_2_ No
   1. If no: Why not?
8. Feel scared
9. Needs to hold it for longer than convenient
10. Feel uncomfortable
11. Others
12. Have something inconvenient happened to you when you go for urination or defecation? 🗖_1_ Yes / 🗖_2_ No
    1. If yes: What have happened
13. Bitten by an animal
14. Someone has been watching
15. Assaulted
16. Others
17. Do you feel that you have to hold it for longer time than convenient?

🗖_1_ Yes / 🗖_2_ No

- 1. If yes: For how long approximately?

1. 1-2 hours
2. 3-4 hours
3. 4-5 hours
4. >6 hours
5. Do you sometimes have to stay home from work because of the toilet situation during some stomach disorder or similar situation? 🗖_1_ Yes / 🗖_2_ No
   1. If yes: How often
6. Once per year
7. 2-5 times per year
8. 6-11 times per year
9. Once per month
10. More than 1 time per month
11. Do you think your working situation would be better if you had access to toilets?

🗖_1_ Yes / 🗖_2_ No

***Part 14 Questions concerning reproduction history and menstrual history***

1. Is your menstrual cycle regular? 🗖_1_ Yes / 🗖_2_ No/ 🗖_3_ Others
   1. If yes: Can you work during your menstrual cycle? 🗖_1_ Yes / 🗖_2_ No/

🗖_3_ Others

- 1. If no: Why not?

1. Lack of toilets
2. No access to dispose sanitary pads
3. Others
4. Do you have access to a place to change sanitary pads during your menstrual cycle? 🗖_1_ Yes / 🗖_2_ No
   1. If yes: Do you go there? 🗖_1_ Yes / 🗖_2_ No
   2. If no: Why not
5. It is too far away
6. It is to dirty
7. It is not private enough
8. Afraid that someone will be aware of you menstrual cycle
9. Others
10. If you don’t have access: How do you manage?
11. Go somewhere else
12. Wait the whole day
13. Stay home from work
14. Others

***Part 15 Questions concerning effects on daily life***

1. Does the toilet situation impact your social life? 🗖_1_ Yes / 🗖_2_ No
   1. If yes: How?
2. Have to stay home more than if there had been toilets
3. Feeling tired
4. UI
5. Others

***Part 16 Questions concerning urinary tract infection***

1. Do you have burning sensation during urination? 🗖_1_ Yes / 🗖_2_ No
   1. If yes: How often?
2. Every day
3. Few days a week
4. Once a week
5. Less than once a week
   1. If yes: For how long?
6. The last week
7. The last month
8. The last 6 months
9. Longer than 6 months
10. Do you have difficulties to hold it? 🗖_1_ Yes / 🗖_2_ No
11. Do you feel difficulty while urinating even if it is needed? 🗖_1_ Yes / 🗖_2_ No
    1. If yes: How often?
12. The last week
13. The last month
14. The last 6 months
15. Longer than 6 months
16. Do you think this problem would have been better if you had better access to toilets? 🗖_1_ Yes / 🗖_2_ No
17. Have you ever been admitted to hospital/medical centre due to urinal or defecation problems? 🗖_1_ Yes / 🗖_2_ No
    1. If yes: Approximately for how many days?.................................................................

***Part 17 Questions concerning menstrual cycle***

1. Do you wash yourself regularly during your menstrual cycle? 🗖_1_ Yes / 🗖_2_ No
2. Do you feel itching around your genitals? 🗖_1_ Yes / 🗖_2_ No
   1. If No, Do you think this problem would have been better if you had better access to toilets? 🗖_1_ Yes / 🗖_2_ No

***Part 18 Questions concerning other health effects***

1. Do you feel any other health problems than the ones mentioned above? 🗖_1_ Yes / 🗖_2_ No
   1. If yes: What kind of health problems?

……………………………………………………………………………………………………….

- 1. If yes: Do you think they may be caused by lack of toilets? 🗖_1_ Yes / 🗖_2_ No

1. Do you think it would be less health problems if you would drink more? 🗖_1_ Yes / 🗖_2_ No
